# Interesting case of ovarian sarcoidosis: The value of multi disciplinary team working

**DOI:** 10.1186/1477-7819-5-38

**Published:** 2007-03-29

**Authors:** Rekha Wuntakal, Rasiah Bharathan, Andrea Rockall, Arjun Jeyarajah

**Affiliations:** 1 Department of Gynaecological Oncology, St Bartholomew's Hospital, London, UK; 2 Department of Radiology, St Bartholomew's Hospital, London, UK

## Abstract

**Background:**

Sarcoidosis of the genital tract is a rare condition. Ovarian manifestation of this disease is rarer still.

**Case presentation:**

The case presented here represents ovarian manifestation of sarcoidosis. At the point of referral to our hospital, based on computerised tomography (CT) ovarian carcinoma was a differential diagnosis. Further magnetic resonance imaging along with CT guided biopsy aided by laboratory study supported a diagnosis of sarcoidosis. Patient responded to medical management by a multidisciplinary team.

**Conclusion:**

The case shows the importance of FNAC and biopsy in case or ovarian masses and multi disciplinary team approach to management.

## Background

Sarcoidosis of the genital tract is a rare condition. Ovarian manifestation of this disease is rarer still. In previously reported cases of genital tract sarcoidosis, the diagnosis was made on surgical specimen. We report the first case of non-surgical management of ovarian sarcoidosis. The successful management of this patient is principally due to the expertise offered by the multi disciplinary team (MDT). Our report highlights the importance of managing complex cases within a MDT environment.

## Case presentation

A 40-year-old para four obese Caucasian woman with two-year history of lower abdominal pain was referred from a peripheral hospital for the further management of bilateral adnexal masses. The computerised tomography (CT) scan of abdomen and pelvis revealed a complex cystic lesion of 7–8 cm in the right adnexum and bilateral ureteric obstruction. All baseline blood tests including haematology and tumour markers (CA 125 -26 IU/ml) were normal. The serum tests showed normal liver function and moderately impaired renal function (creatinine 10–15, urea > 200).

She had a complex medical history including obstructive sleep apnoea, asthma, glaucoma, hypothyroidism, hypertensive cardiomyopathy and bipolar disorder; registered disabled. She also had a copper intra uterine contraceptive device *in situ *for the past three years. Given her body habitus (BMI 42), abdominal and pelvic examination was difficult and non-specific. The only finding of relevance was bilateral adnexal fullness.

She was managed within the gynaecological oncology multi disciplinary framework (MDT). On reviewing the CT films the features noted include bilateral basal pleural thickening, bilateral hydronephrosis, retroperitoneal lymphadenopathy associated with retroperitoneal fibrosis and bilateral adnexal cystic masses. There was a small volume of ascites.

**Figure 1:**
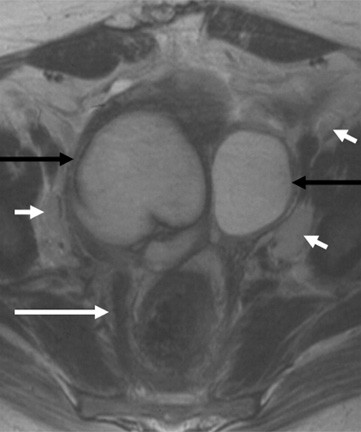
MRI axial T2W images of the pelvis. Bilateral adnexal cystic masses are demonstrated (black arrows). The cyst wall on the right side is thick and irregular. There is marked thickening of the mesorectal fascia (long white arrow). Multiple prominent nodes are demonstrated along the pelvic sidewalls, which are very high in signal intensity on T2W (short white arrows).

Magnetic resonance imaging (MRI) was performed for further characterisation of the adnexal masses. The right adnexal complex cystic lesion measured 8 × 7 × 7 cm in diameter and had a thick wall (up to 2 cm), which was irregular. There were several septations, some of which measured 3 – 4 mm. There were no enhancing papillary formations, although the wall and septa enhanced. The appearance suggested an ovarian lesion with possible involvement of the Fallopian tube. The left adnexal cyst measured 5 × 6 × 4 cm, had a smooth wall and contained a single thin septation, consistent with a benign ovarian cyst. Both cysts contained fluid, which was low on T1 and high on T2, with no evidence of fat or blood. Surrounding the cysts posteriorly, the fat was streaky in appearance and there was marked thickening of the adjacent fascial planes, with thickening of the mesorectal fascia up to 1 cm. The fascial thickening extended up the pre-sacral space and along the posterior aspect of the pelvic sidewalls, resulting in the obstruction of the ureters. Enlarged lymph nodes (up to 1.8 cm in short axis) were present along both pelvic sidewalls, which were striking due to the very high T2 signal intensity (Figure 1). Nodes were also seen in the inguinal and para-aortic regions. Although the imaging appearances could not rule out an ovarian neoplasm, the features noted above were suggestive of an inflammatory process. In view of the woman's medical history and clinical status the MDT recommendation was clinical review and imaging guided biopsy of lymph nodes.

The woman underwent CT guided right inter iliac node biopsy, following an unyielding biopsy of inguinal nodes. Histology revealed non-caseating granulomatous lymphadenitis with negative stains for acid-fast bacilli and fungi. There was no foreign body seen. Given the histological findings our attention was directed towards an inflammatory process. She was subsequently referred to respiratory physicians, with a working diagnosis of sarcoidosis. She was also referred to urologists for the management of retroperitoneal fibrosis and bilateral hydronephrosis. Abdominal pain prompted insertion of bilateral JJ ureteric stents. The physicians initiated a (step-down) course of prednisolone. Symptomatic improvement was observed but the renal function did not change. Repeat imaging demonstrated resolution of hydronephrosis.

**Figure 2:**
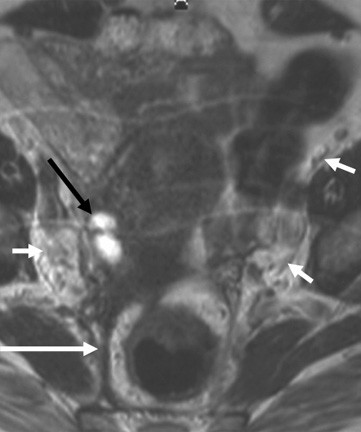
MRI axial T2W image of the pelvis following treatment for sarcoidosis. The left adnexal cyst has resolved. There is a small residual cyst on the right (black arrow). There is marked decrease in the thickening of the mesorectal fascia (white arrow). The lymph nodes appear normal (short white arrows).

The woman was reviewed again clinically after the course of steroids. During this interval the physicians ordered an autoantibody screen as autoimmune disease can co-exist with sarcoidosis; the screen was negative (Rheumatoid factor, anti-DNA antibody and antinuclear antibody). On examination there was no evidence of any palpable lymphadenopathy. An ultra sonographic examination six months later demonstrated a bulky uterus and reduction in the size of the right adnexal cyst. Repeat imaging (MRI axial T2W image) a further two months later demonstrated resolution of the left adnexal cyst. There was a small residual cyst on the right and marked decrease in the thickening of the mesorectal fascia. The lymph nodes appeared normal (Fig 2). The retroperitoneal fibrosis on imaging had resolved. Her CA125 remained normal. Currently the patient is under joint care between the respiratory and renal physicians with symptomatic improvement.

## Discussion

Boeck in Norway first identified sarcoidosis over 100 years ago. Sarcoidosis was originally called Boeck's disease. It was defined in 1960 as a systemic granulomatous disease of undetermined aetiology and pathogenesis [1].

It has high prevalence in European countries (Sweden and Denmark). The UK prevalence rate is 20 per 100 000, and the incidence increases from north to south [2]. The primary targets include lungs, lymph nodes, liver and spleen. Ovarian sarcoidosis presenting as bilateral adnexal masses is extremely rare whether as a component of systemic disorder or as an isolated finding [3]. Both the presentation and the findings can mislead a clinician towards sinister diagnosis. The aetiology of sarcoidosis remains unclear. Onset is most common between the ages of 20 and 40 years and usually presents with bilateral hilar lymphadenopathy and pulmonary infiltration. Clinical manifestations such as amenorrhoea, menorrhagia, postmenopausal bleeding and erosion of the cervix have also been reported [2]. On basis of histology and clinical as well as radiological response of the woman to first line anti-sarcoid treatment, the case described here fulfils the criteria for the diagnosis of sarcoidosis.

Only a few cases of sarcoidosis of the female genital tract have been reported, and Winslow et al [4] feel this may be due to under reporting of the true incidence. The most common site of involvement of the female reproductive system is the uterus. Ovarian sarcoidosis is an extremely rare condition and is known to mimic ovarian malignancy. To date only seven cases of ovarian involvement have been reported in the English language literature [3-9]. Four of these cases revealed uterine involvement as well on pathological examination. Ours is the first case with a non-surgical approach in a patient with ovarian sarcoidosis.

In the reported cases to date, the age of the patients ranged between 32 years and 72 years. The gynaecological problems in these women were postmenopausal bleeding, menorrhaegia, cervical carcinoma in situ, abdominal pain and abdominal distension. Three of the seven patients had prior history of documented sarcoidosis and it was an incidental finding in the remaining cases. In our case report, the main complaint was abdominal pain with adnexal masses leading to cancer referral.

Preoperatively in one case, the CT scan of the abdomen revealed enlarged lymph nodes in para aortic and mesenteric region and in the second case the mediastinal nodes were enlarged [3,5] Clinically and radiologically (ultrasound and CT scan) a mass was noted in the pelvis in three cases prior to surgical treatment [3,5,9] In our case report the retroperitoneal nodes were also enlarged.

In three reported cases, women underwent exploratory laparotomy with a provisional diagnosis of ovarian malignancy but the histological diagnosis was sarcoidosis. The CA-125 was between 248–477 IU/ml [3,5,6]. Postmenopausal bleeding, menorrhaegia and cervical carcinoma in situ accounted for the other four women who underwent hysterectomy and bilateral salpigo-ophorectomy. The pathological examination of the specimen suggested sarcoidosis[4,7-9].

More obscure causes of the genital tract granulomas include coccidiomycosis, lymphogranuloma inguinale, foreign body reaction and leprosy. Bacteriological proof is essential to differentiate these from sarcoidosis. These can be differentiated by appropriate history, special staining of tissues and microscopic findings. [5,9]

There are no specific radiological findings in the literature to describe ovarian sarcoidosis. In the opinion of the radiologists, an intra abdominal (and thoracic) inflammatory process rather than malignancy was more likely to explain the findings. The striking feature on the MRI of this woman was the presence of marked thickening of fascial planes, which is not typical of ovarian cancer. The serum CA 125 level is raised in 80% of epithelial ovarian cancer. Given the normal level of CA 125 and unusual radiological findings it reassured us that this was not obviously an ovarian malignancy.

Following a CT guided lymph node biopsy an empirical diagnosis of sarcoidosis was made on the basis of clinical and laboratory findings with histological support. This woman was managed conservatively, given the woman's poor anaesthetic profile. A percutaneous biopsy of the lymph node facilitated arrival at the diagnosis with minimal morbidity.

Although White *et al *[3] described coexistence of mucinous cystadenoma of the ovary and ovarian sarcoidosis. In our case given the resolution of pelvic mass with steroids it was unlikely to be cystadenoma.

Subsequent clinical review and repeat imaging after steroid therapy demonstrated resolution of the left adnexal cyst. The lymph node biopsy together with follow-up imaging post treatment offered evidence to suggest that the process was likely to be sarcoidosis. Given the clinical and radiological improvement of the patient, following MDT discussion the woman was satisfactorily discharged from our care to be monitored by physicians.

The case in point not only illustrates a rare manifestation of sarcoidosis affecting the ovary but also highlights the importance of managing such complex women in a MDT environment to optimise care. MDT approach can function as an ideal conduit for the management of complex patients with complex adnexal masses. The presence of an expert diagnostic radiologist in this setting is vital in determining the subsequent modality of care. Although preoperative imaging of adnexal masses is routine but where doubt exists regarding the diagnosis, a simple preoperative imaging guided biopsy of suitable tissue can help avoid major surgery and the attending risks. The resources of the MDT have ensured appropriate care and optimum outcome for this woman.

## Conclusion

We describe a case of ovarian sarcoidosis, which is a rare clinical entity. The presentation can mimic that of carcinoma. Therefore optimum management of such patients should be delivered within a MDT environment.

## Competing interests

The author(s) declare that they have no competing interests.

## Authors' contributions

**RW **and **RB **– Primary co-authors in producing and revising the manuscript.

**AR **– interpreted and correlated imaging data with text narrative. Contributed to concept development and revised manuscript.

**AJ **– Concept development and authority over final draft

All authors have read and approved the final manuscript.
